# Topoisomerase I poisons-induced autophagy: Cytoprotective, Cytotoxic or Non-protective

**DOI:** 10.1080/27694127.2022.2155904

**Published:** 2022-12-25

**Authors:** Ahmed M. Elshazly, Polina A. Wright, Jingwen Xu, David A. Gewirtz

**Affiliations:** a Department of Pharmacology and Toxicology, Virginia Commonwealth University, Massey Cancer Center, 401 College St., Richmond, VA 23298, USA; b Department of Pharmacology and Toxicology, Faculty of Pharmacy, Kafrelsheikh University, Kafrelsheikh 33516, Egypt; c School of Pharmacy, Guangdong Pharmaceutical University, Guangzhou 510006, China

**Keywords:** Irinotecan, Topotecan, Autophagy, Cytoprotective, Cytotoxic, Non-protective

## Abstract

Topoisomerase I inhibitors represent a widely used class of antineoplastic agents that promote both single-stranded and double-stranded breaks in the DNA of tumor cells, leading to tumor cell death. Topotecan and irinotecan are the clinically relevant derivatives of the parent drug, camptothecin. As is the case with many if not most anticancer agents, irinotecan and topotecan promote autophagy. However, whether the autophagy is cytotoxic, cytoprotective, or non-protective is not clearly defined, and may depend largely upon the genetic background of the tumor cell being investigated. This review explores the available literature regarding the nature of the autophagy induced by these clinically utilized topoisomerase I inhibitors in preclinical tumor models with the goal of determining whether the targeting of autophagy might have potential as a therapeutic strategy to enhance the antitumor response and/or overcome drug resistance.

## 1. Introduction

This manuscript is one in a series of papers that explore the role of autophagy in the response to therapeutic modalities in tumor cells. Our previous publications covered radiation [1], cisplatin [2], microtubules poisons [3], hormonal therapies in estrogen positive breast cancer [4], as well as PARP inhibitors [58].

## 2. Overview of Autophagy

Autophagy, derived from Greek, and meaning “self-eating”, is a distinct self-degradative process designed to maintain cellular and metabolic hemostasis via the elimination of damaged organelles, intracellular pathogens, as well as providing energy upon nutrient deprivation [5]. Autophagy is generally considered as a survival mechanism [6]. In mammalian cells, there are three different types of autophagy: microautophagy, macroautophagy, and chaperone-mediated autophagy [7]. In microautophagy, cargo is captured by lysosomal membrane protrusions while chaperone-mediated autophagy differs in that it does not utilize membranous structures to sequester cargo; instead, chaperones identify cargo proteins [7], which are directly transported into the lysosomal membrane [8]. Most studies in the literature are focused on macroautophagy, which is commonly referred to as autophagy.

One of the earliest events in macroautophagy is the formation of the phagophore, a double membrane structure that encloses damaged cytoplasmic components [9]. The phagophore edges extend, elongate and engulf portions of the cytoplasm, then fuse together forming the autophagosome that later fuses with lysosomes, resulting in autolysosome formation, which is responsible for cargo degradation [9]. This multistep process is tightly regulated by several highly conserved autophagy (ATG) proteins as well as a number of molecular pathways including the phosphatidylinositol 3-kinase/mammalian target of rapamycin (PI3K/mTOR) and AMP-activated protein kinase (AMPK) signaling pathways [10].

Autophagy is one of the basic processes involved in cellular homeostasis, and consequently defective autophagy has been associated with a number of pathologies such as cardiac disease, as well as neurodegenerative conditions including Alzheimer’s, Huntington and Parkinson’s disease [11]. Autophagy is also associated with cancer therapies, wherein autophagy has been identified to play four different roles, specifically cytoprotective, cytotoxic, cytostatic, and non-protective functions [12], although the cytostatic function has been largely overlooked. Our own laboratory as well as others [13] have reported a specialized cytotoxic form of autophagy which either kills cells on its own or acts to trigger apoptosis; for example, we reported that vitamin D (or the vitamin D analog, EB 1089) in combination with radiation promotes a cytotoxic form of autophagy in breast tumor cells [12, 14, 15]. Another form is cytostatic autophagy, which contributes to growth inhibition without promoting apoptosis and may be associated with senescence (although the relationship between autophagy and senescence is complex and inconsistent in different models). Dou et al. have reported that ivermectin induces cytostatic autophagy in breast cancer cells with growth suppression without apoptosis, where autophagy inhibition via Beclin 1 or ATG-5 knockdown restores tumor cell growth [16]. Our laboratory reported that vitamin D (or the vitamin D analog, EB 1089) in combination with radiation resulted in a significant growth inhibition in non–small cell lung cancer cells with no cell killing [12].

A lesser appreciated form is that of non-protective autophagy, where autophagy inhibition by pharmacological or genetic approaches fails to contribute to cell death. Recent studies from our laboratory studying the effect of the Fulvestrant plus Palbociclib combination in the MCF-7 breast tumor cell line found that pharmacological autophagy inhibition utilizing CQ or bafilomycin or genetic autophagy inhibition by ATG-5 knockdown produced, at best, a modest sensitization of the cells to the combination effects (manuscript in review), indicating the largely non-protective role that autophagy plays under these experimental conditions.

The most widely studied form with potential for therapeutic applications is cytoprotective autophagy, which is considered a survival mechanism whereby cancer cells evade the cytotoxicity of many anti-neoplastic therapies, and ultimately leading to the development of resistance. In just one of many examples in the literature, Circu et al. [17] showed that CQ increased sensitivity to cisplatin via autophagy inhibition in a cisplatin-resistant A549 NSCLC cell line (A549/cisplatin)[2, 17]. Multiple clinical trials have been initiated in different types of cancer that evaluate the potential use of autophagy inhibitors such as hydroxychloroquine in combination with chemotherapeutic agents.

## 3. Clinically used topoisomerase I inhibitors

**Figure 1.**
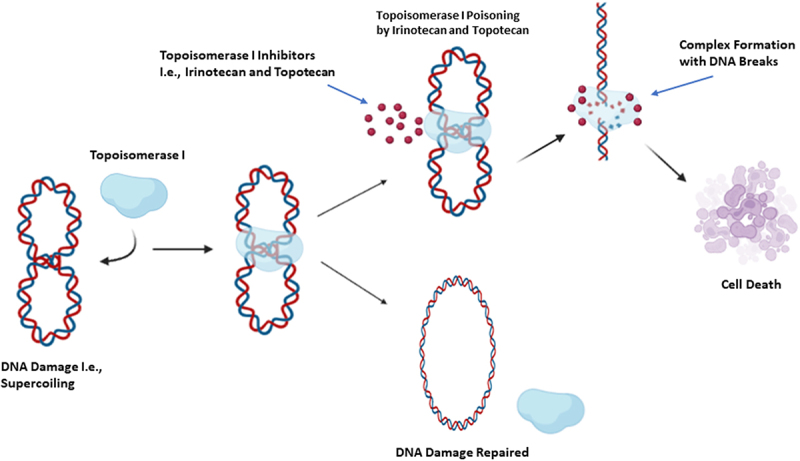
The mechanism of action of topoisomerase I inhibitors. Irinotecan and topotecan exert their antitumor effects via covalent binding with topoisomerase I, resulting in Top I- Irinotecan/Topotecan-DNA complex formation. This complex generates single and double-strand breaks in DNA, leading to cell death.

DNA’s compact and supercoiled nature requires distinct modifications during vital cellular processes such as transcription, replication, and repair. This modification is achieved via DNA strand cleavage, strand passage and re-ligation mediated by DNA topoisomerases. DNA topoisomerases are a class of enzymes that cleave the DNA sugar-phosphate backbone without altering its chemical composition; the two most widely discussed classes in the literature are Type I (Top I) and Type II (Top II) topoisomerases [18]. Top I generates DNA single-strand breaks to permit the relaxation of torsional stresses before the re-annealing step while Top II generates DNA double-strand breaks to allow the passage of the intact duplex through the gap before rejoining [18, 19]. A number of antineoplastic agents have DNA topoisomerases I or II as their molecular targets [20]. Among these, camptothecin is a plant alkaloid extracted from the Chinese tree *Camptotheca acuminata* that poisons Top I; however, the clinical development of camptothecin was discontinued due to the significant adverse effects observed as well as its low therapeutic index [21]. The primary clinically-tolerable and water-soluble derivatives of camptothecin are topotecan and irinotecan [21]. These compounds contain the same ring structure involving a lactone moiety which is necessary for anti-cancer activity [22]. Like camptothecin, these compounds poison Top I by formation of Top I –DNA complexes in a manner that prevents the re-ligation of DNA (Figure 1).

Irinotecan is a prodrug that acts at the S and G2 phases of the cell cycle [23] via topoisomerase I -targeting. Irinotecan becomes activated through the actions of a carboxylase-converting enzyme to its biologically active form, SN-38, a metabolite with higher topoisomerase I -inhibiting properties than Irinotecan [24, 25]. Irinotecan is utilized in the clinical setting in the

treatment of colorectal cancer [26], pancreatic cancer [27, 28] as well as small cell lung cancer [29, 30]. Topotecan has the same mode of action as camptothecin and irinotecan, exerting its cytotoxic effects during the G1 and S-phases [31]. In the clinic, Topotecan is utilized in the treatment of ovarian cancer, small cell lung cancer as well as cervical carcinoma [28, 30].

Although irinotecan and topotecan have proven to be effective antineoplastic agents, both de novo and acquired resistance remain persistent clinical problems, as is the case for many cancer chemotherapeutic agents [32]. A number of mechanisms underlying resistance have been identified; these include Top I alterations [33], changes in cellular response to the Top I–drug interaction, overexpression of an SN-38 inactivator [30, 34] and cellular efflux. A focus of this article is whether autophagy also confers resistance to topoisomerase I inhibitors/poisons and consequently whether targeting autophagy might provide a therapeutic advantage in association with the clinical use of irinotecan and topotecan.

Topoisomerase I inhibitors have definitely been shown to trigger autophagy. For example, Chiu et al. [35] showed that camptothecin induced autophagy in H1299 and H460 non-small cell lung cancer cell lines; inhibition of the autophagy with 3-methyladenine increased camptothecin induced DNA damage as well as drug cytotoxicity. This is an example of cytoprotective autophagy.

**Figure 2.**
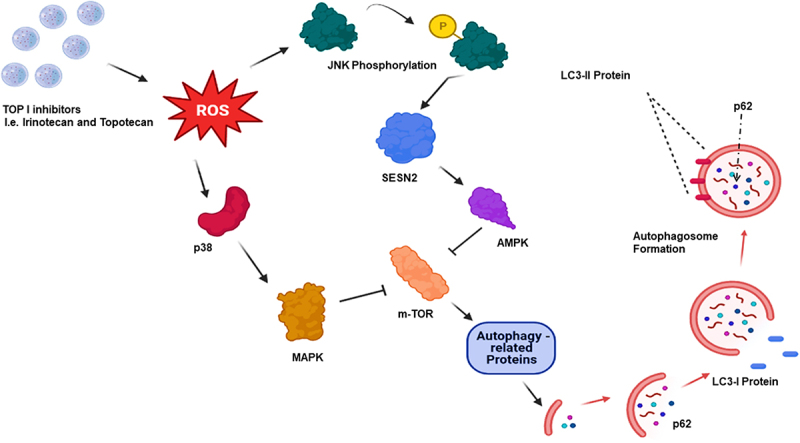
Top I inhibition triggers autophagy via m-TOR suppression. Topoisomerase I inhibition by irinotecan or topotecan generates reactive oxygen species (ROS), which drives the activation of JNK/AMP and p38/MAPK. JNK phosphorylation activates SESN2, followed by AMPK. p38 activation via ROS upregulates MAPK. These two pathways converge on m-TOR, where m-TOR inhibition triggers autophagic flux.

Irinotecan has been shown to generate reactive oxygen species (ROS), which activate the JNK (Jun Nuclear Kinase) and p38-MAPK (mitogen-activated protein kinase) pathways [36]. Topotecan has also been shown to generate ROS, leading to JNK phosphorylation and activation of the p-JUN-SESN2-AMPK cascade [37, 38]. These topoisomerase I inhibitor actions converge on mTOR inhibition, activating autophagy related proteins such as ULK1, ATG13, ATG4L, as well as AMBRA1, ultimately promoting autophagy [39] (Figure 2). In this review, we explore the relationship(s) between autophagy and the clinically used Top I inhibitors, irinotecan and topotecan, in efforts to determine whether autophagy inhibition has the potential to serve as an effective therapeutic strategy.

## 4. Irinotecan and SN-38 induced autophagy

A relatively limited number of papers have been published regarding the potential role of autophagy in response to either irinotecan or its active metabolite, SN-38. Interestingly, there is little consensus as to the nature and role of the autophagy, which may be related to the different cell lines used in these studies. Chen et al. [40] reported that treatment with the irinotecan metabolite, SN-38, induced autophagy in LOVO and HCT116 colorectal cancer cell lines, as evidenced by LC3-II elevation as well as p62/SQSTM1 degradation, standard indicators of autophagy. However, there were no evident differences in SN-38-induced cytotoxicity in the LOVO, HCT116, and SW1116 cell lines with autophagy inhibition utilizing the pharmacological agent, 3-methyl adenine (3-MA), when compared to the controls treated with SN-38 alone. Similarly, autophagy inhibition via genetic approaches utilizing ATG5-targeted siRNA did not enhance SN-38- mediated cytotoxicity in either LOVO or HCT116 cell lines [40]. These findings suggest that the autophagy was functionally *nonprotective*. In contrast, and unexpectedly, the combination of SN-38 with another pharmacologic autophagy inhibitor, chloroquine (CQ), resulted in a synergistic cytotoxic effect *in vitro* in the SW116, LOVO, and HCT116 cell lines; moreover, the inclusion of CQ resulted in reduced tumor volume *in vivo* using xenograft models of athymic BALB/c nude mice implanted with the SW1116 and LOVO cell lines. In reconciling these findings, the authors concluded that the sensitization observed was independent of CQ function as an autophagy inhibitor and largely dependent on ROS production and several other apoptotic pathways [40]. Furthermore, using SW480 and HT-29 colorectal cancer cell lines overexpressing mutant p53, the SN-38 and CQ combination treatment *did not* duplicate the synergistic cytotoxic effect; instead the combination index suggested a slightly antagonistic effect. Taken together, these results suggest that the autophagy induced by SN-38 in colorectal cancer cell lines was largely *nonprotective*; however as is evident from further work described below, the status of p53 likely influences the nature of the autophagy.

Interestingly, Chen et al. [41], using western blotting, lysotracker staining and immunofluorescence microscopy, reported that the *irinotecan-resistant* LOVO colon cancer cell line showed higher expression than the parental cell line of the autophagy promoting proteins Beclin-1, Atg3, Atg7, LC3-I, LC3-II as well as ULK1. In addition, the resistant cell line showed increased numbers of lysosomes and lysosomal activity [41], indicating that the basal level of autophagy is higher in the irinotecan-resistant cells than the drug-sensitive cells. Furthermore, treatment with QNZ (N4-[2-(4-phenoxyphenyl)ethyl]-4,6-quinazolinediamine), an NF-κB activation inhibitor [41], resulted in a dose-dependent reductions in resistant cell survival, reductions in p-NF-κB-p65^Ser536^, Beclin-1, Atg3, Atg5, Atg7, LC3, ULK1, as well as different metastatic markers [41], suggesting that the activation of NF-κB signaling pathway may be one mechanism whereby *irinotecan-resistant* LOVO cell line undergo autophagy as well as metastasis. However, this work did not define the functionality of the autophagy.

Tamura et al. [42] showed that treatment with the irinotecan metabolite, SN-38, combined with autophagy inhibitors including 3-methyladenine or bafilomycin A1, significantly *diminished* the SN-38 mediated cytotoxicity in the HSC-4 OSCC (human oral squamous cell carcinoma) cell line, indicative of the *cytotoxic* function of autophagy. SN-38-mediated cytotoxicity, however, was not influenced by pharmacological autophagy inhibition in the HSC-2 OSCC cell line [42], indicative of *nonprotective* autophagy, and supporting the premise that the function of autophagy is not only cancer-specific but is also likely cell line-specific.

Zhu et al.[36] showed that irinotecan treatment in MGC803 and SGC7901 gastric cancer cells lines triggered apoptosis, as evidenced by the increased caspase 3 and cleaved PARP levels increased ROS levels, as well as suppressed cellular growth. Irinotecan also induced autophagy, as indicated by the upregulation of LC3-II and Beclin-1 levels as well as p62/SQSTM1 degradation. Autophagy inhibition using the pharmacological agents 3-MA and CQ, resulted in *increased* proliferation in the irinotecan-treated cells, indicating that autophagy blockade could diminish the antitumor effects of irinotecan, and suggesting that here the autophagy was *cytotoxic* in function. Using a DCF-DA assay kit as a readout, these authors reported a rise in ROS levels after treatment with irinotecan and that co-treatment with the antioxidant, NAC, resulted in a reduction in ROS accumulation, decreased LC3-II levels, increased cell viability, and decreased caspase 3 and cleaved PARP compared to irinotecan alone, demonstrating that ROS was involved in the growth inhibition and irinotecan-induced autophagy. Additionally, p-JNK and p-p38 western blot levels were attenuated upon treatment with a combination of irinotecan and NAC, indicating that JNK- and p38-MAPK signaling pathways are moderated through ROS and play a role in irinotecan-induced autophagy.

In additional studies utilizing a xenograft model where SGC7901 cells were injected subcutaneously into BALB/c nude mice, irinotecan treatment resulted in increased LC3-II levels, cleaved caspase 3, cleaved PARP as well as increases in p-JNK and p-p38 in tumor tissues; however, 3-MA dramatically attenuated these effects, suggestive of a *cytotoxic* function of autophagy. Furthermore, irinotecan treatment alone markedly reduced the tumor volume compared to the combination of irinotecan with 3-MA, supporting the conclusion that the autophagy was mediating drug action and was therefore *cytotoxic* in function. Additionally, irinotecan combined with 3-MA reduced (TUNEL)-positive tumor cells and increased the proportion of Ki67-positive cells in tumor tissues as compared to the irinotecan alone [36], further consistent with the conclusion that autophagy may play a *cytotoxic* role in MGC803 and SGC7901 gastric cancer cells lines. However, a limitation of these studies is the absence of genetic approaches for autophagy inhibition.

Recently, Zhang et al. [43] studied toosendanin (TSN), a triterpenoid extracted from the root bark of *Melia toosendan* Sieb. *Et* Zucc, utilizing triple-negative breast cancer cell lines including MDA-MB-231 and MDA-MB-436 cells. They reported that TSN has the ability to inhibit late-stage autophagic flux, as evidenced by LC3-II and p62/SQSTM1 accumulation, GFP-LC3 and RFP-GFP-LC3 fluorescence as well as the unchanged LC3-II levels after bafilomycin A1 co-treatment. Moreover, they showed that TSN did not inhibit lysosome and autophagosome fusion, as confirmed by examining RFP-GFP-LC3 co-localization and the late endosomal and lysosomal membranes marker LAMP1; however, TSN increased the pH of the lysosome and compromised the lysosomal proteolytic function as visualized through fluorescence (late-stage autophagy). Importantly, they showed that SN-38 treatment induced autophagy based on a dose- dependent increase in LC3-II levels, decreased p62/SQSTM1 levels as well as by utilizing RFP-GFP-LC3, which showed a dramatic red-only puncta elevation after treatment with SN-38. TSN sensitized the breast cancer cells to SN-38 based on cell morphology changes, the LDH release assay, the MTT assay, Annexin V-FITC/PI staining, increased caspase 3 levels by western blotting, and increased ROS release, with clear evidence for autophagy inhibition confirmed by the RFP-GFP-LC3 reporter. Here, then, the autophagy appeared to take on its more classical *cytoprotective* function.

These researchers also investigated the combination of TSN and irinotecan *in vivo* in a xenograft model of nude mice injected subcutaneously with MDA-MB-231 cells expressing RFP-GFP-LC3. Irinotecan in combination with TSN significantly reduced tumor growth and tumor weight as well as promoting cleavage of caspase 3 as compared to treatment with irinotecan alone. Moreover, TSN treatment blocked the irinotecan-induced autophagy in a dose-dependent manner, as evidenced by increased LC3-II and p62/SQSTM1 levels compared to irinotecan treatment alone, with increased RFP-GFP-LC3 yellow puncta indicating interference with autophagosome maturation [43]. These results are consistent with the *cytoprotective* function of irinotecan/SN-38 mediated autophagy in a triple negative breast cancer cell model.

Paillas et al. [44] reported that SN38 induced autophagy in the HCT116-TP53 KO colon cancer cell line based on increased LC3-II levels and the appearance of large double-membrane cytoplasmic vacuoles. Autophagy inhibition by transfection with siRNA for ATG5 and ATG7 increased SN-38 cytotoxic effects as compared to SN-38 alone, indicating a *cytoprotective* role for autophagy mediated by SN-38 in this cell line. A minor concern with these studies was the limited number of autophagy markers utilized and the absence of experiments involving pharmacological autophagy inhibition.

Paillas et al. [44] further investigated the importance of MAPK14 in autophagy in the HCT116-TP53 KO colon cancer cell line, showing that the suppression of MAPK11, MAPK12, or MAPK13 via shRNA had no effect on SN38 toxicity; in contrast, MAPK14 silencing increased the SN-38 mediated cytotoxicity as evidenced by a lower IC_50._ MAPK14-overexpressing cells showed non-senescence-dependent slowing in growth, confirmed by negative β-galactosidase staining, as well as characteristics of autophagy induction, including increased LC3-II, double membrane vacuoles detected by electron microscopy, and enhanced punctuated GFP-tagged LC3 fluorescence. Interestingly, MAPK14 downregulation via shRNA prevented SN-38-induced autophagy based on the lack of induction of LC3-II. Furthermore, autophagy inhibition via pharmacologic approaches using bafilomycin A1 or 3-MA or by genetic suppression with siATG5 and siATG7 decreased cell viability in MAPK14 overexpressing cells treated with SN-38 in comparison to the controls treated with SN-38 alone, emphasizing the contribution of MAPK14 in SN38-mediated *cytoprotective* autophagy [44].

Although a number of the cited studies indicated that the autophagy induced by irinotecan/SN-38 autophagy was cytoprotective, there are also clear examples where the autophagy was either cytotoxic or non-protective, depending on which cancer cell line was used. Given this diversity and unpredictability of the response, it appears unlikely that autophagy targeting would present a consistent and clinically relevant therapeutic strategy in patients whose tumors are being exposed to irinotecan or its active metabolite, SN-38.

## 5. Topotecan-induced autophagy, p53 and the autophagic switch

The potential involvement of autophagy in protecting tumor cells from the cytotoxicity of topotecan has been explored in only a limited number of studies. In the A549 non-small cell lung cancer cell line, Wang et al.[45] showed that topotecan induced autophagy, as evidenced by pYFPLC3 expression by fluorescence microscopy, acridine orange staining, increased LC3II as well as p62/SQSTM1 degradation. They further reported that upon combining CQ with topotecan, there was a significantly greater reduction in cell viability than for either drug alone accompanied by increased apoptosis. ATG-5 knockdown mediated by siRNA also enhanced topotecan cytotoxicity [45], supporting the conclusion that autophagy induced by topotecan may have a *cytoprotective* role.

It is long established that p53 acts as the guardian of the genome and is a “gate keeper” against tumorigenesis. Not only does p53 play an important regulatory role in tumor cell proliferation, cell cycle and apoptosis, it is also associated with cellular autophagy and is closely linked to cellular sublocalization. It was shown that p53 located in the nucleus promotes autophagy under stress. In contrast, in unstressed cells, cytosolic p53 inhibits autophagy [46, 47]. In the nucleus, p53 induces autophagy by regulating the mTOR pathway in a transcription-dependent manner, as well as transcriptionally regulating key ATG genes [48]. In general, patients whose tumors exhibit p53 mutations or deletions tend to have a poorer prognosis [49]. However, interestingly, topotecan is not uniformly sensitive to the status of p53 in different tumor types. Kaina’s laboratory [50] found that p53 mutant glioblastoma U138 cells showed greater sensitivity to topotecan than p53 wild-type glioblastoma U87 cells. Consistent with this observation, the p53 inhibitor pifithrin-α and p53 siRNA increased topotecan-induced cell death in p53 wild-type U87 cells [50]. However, in colon cancer, Li et al. [51] found that there was no difference in the sensitivity of colon cancer cells with differing p53 status to topotecan.

To further investigate whether p53 state sensitivity to topotecan is associated with autophagy, Li [42] et al. detected topotecan-induced autophagy in HCT116, LS-174T and HT29 cells by accumulation of YFP-LC3 foci, LC3-II accumulation by immunoblotting, and p62/SQSTM1 degradation. Autophagy inhibition mediated by beclin1 and ATG5 siRNA increased topotecan-induced tumor cell death in p53 wild-type HCT116 and LS-174T cell lines. Autophagy inhibition via a pharmacological approach utilizing CQ showed a similar trend to the genetic knockdown studies, with an increase in topotecan-mediated cell death. Consequently, the autophagy was clearly *cytoprotective* in function in p53 wild-type colon cancer cells. Interestingly, however, in contrast to the outcomes observed in the p53 wild-type cell lines, autophagy inhibition in p53 mutant HT29, SW620, and SW480 cell lines using CQ *blocked* the cell death induced by topotecan treatment, indicating that the autophagy was *cytotoxic* in function in p53 mutant colon cancer cells. Even more unexpectedly, in HCT116 p53-/- cells, treatment with CQ reduced sensitivity to topotecan, while ATG5 shRNA had no impact on the sensitivity of HCT116 p53-/- cells to topotecan [51]. Taken together. these results were indicative of a topotecan-induced *cytoprotective* role for autophagy in p53 wild type colon cancer cell lines; however, the evidence for *cytotoxic and/or nonprotective* functions of autophagy in the cell lines lacking functional p53 is reflective of what we have termed the “autophagic switch” [52], where one form/function of autophagy is converted to another when p53 function has been compromised.

Li et al. [51] also investigated the effect of inhibition of topotecan-mediated autophagy using the pharmacologic autophagy inhibitor, CQ, *in vivo*, in a xenograft model by subcutaneous injection of HCT116 p53^+/+^ and HCT116 p53^−/−^ tumor cells in athymic nude mice. Topotecan in combination with CQ increased the anti-tumor effects in the HCT116 p53+/+ xenograft model over that of topotecan alone; in contrast the inclusion of CQ seemed to impair the anti-tumor activity of topotecan in the HCT116 p53^−/−^ model. Here again we observe an autophagic switch that is p53 dependent. Combining compound C (a potent and selective AMPK inhibitor that suppressed autophagy) with topotecan *in vivo* showed a similar trend to that of CQ, with increasing topotecan-antitumor effects in HCT116 p53^+/+^ tumor cells but not in the p53^−/−^ xenograft model [51].

It should be emphasized that the phenomenon of the “autophagic switch” relating to the status of p53 status is not an isolated observation in topotecan-treated cells. For example, bafilomycin A1 increased the sensitivity of Crocin (the bioactive molecule of saffron) treated p53^+/+^ HCT-116 cells but had no effect on p53^−/−^ HCT-116 cells (switching from cytoprotective to non-protective autophagy) [53]. In our own work involving the combination of pharmacological or genetic autophagy inhibition with cisplatin, p53 wild-type H460 cells showed *non-protective* autophagy in response to cisplatin treatment whereas p53 knockdown H460 cells exhibited *cytoprotective* autophagy [54]. However, it is worth noting from the above examples that although p53 does intrinsically correlate with autophagy, p53 status does not imply that autophagy must have a particular function.

It remains uncertain as to why p53 regulates the role of autophagy and subsequently affects sensitivity to chemotherapeutic drugs. In the case of topotecan, what is known thus far is that topotecan treatment causes a dose-dependent elevation in p53 levels, increased expression of the p53 target, sestrin 2, increased phosphorylation of AMPK, an upstream regulator of autophagy [55] as well as inhibition of the mTORC1 pathway in the p53 wild-type HCT116 and LS174T cell lines. Moreover, topotecan when combined with siRNAs targeting p53 or sestrin 2 abrogates the activation of AMPK and LC3-II accumulation, suggesting that p53 regulates the autophagy induced by topotecan via sestrin 2 and AMPK activation [51]. Furthermore, AMPK inhibition via compound C increases topotecan cytotoxicity in the p53 wild-type (HCT116 and LS174-T) cell lines, but does not sensitize the mutant p53 (HT29, SW620 and SW480) cell lines. At the same time, inhibition of topotecan-mediated AMPK activation blocks autophagy induction as well as removing mTOR inhibition [51], indicating that topotecan mediated autophagy depends on AMPK and its downstream inhibition of mTORC1.

Collectively, while a cytoprotective role of topotecan-mediated autophagy and its potential targeting as a possible therapeutic strategy to increase the effectiveness of topotecan-based therapy might have utility in p53 wild-type cells, this does not appear to be the case in tumor cells lacking functional p53.

## 6. Conclusions

Topoisomerase 1 inhibitors, specifically irinotecan and topotecan, clearly have clinical utility as antineoplastic agents; however, as the case with other anticancer drugs, the development of resistance may interfere with their therapeutic efficacy. Among the different molecular mechanisms of resistance, a number of publications have identified autophagy in response to irinotecan/SN-38 or topotecan with the possibility of its targeting. An additional connection between autophagy and topoisomerase I has recently been demonstrated in the literature with the identification of a new autophagy receptor, TEX264, that may play a role in repairing topoisomerase 1-DNA adducts [56, 57]. As summarized in Table 1, the function of autophagy in response to irinotecan or SN-38 is not consistently that of cytoprotection, which would be required if autophagy inhibition could be developed as a therapeutic strategy. Furthermore, p53 status as well as the autophagic switch further complicate this potential approach to enhancing drug sensitivity. Finally, the fact that we cannot measure autophagy in patient tumors or determine whether the autophagy has actually been inhibited using hydroxychloroquine or chloroquine, which may produce their effects independently from autophagy, raises the question as to whether autophagy can be a valid target to sensitize tumor cells to irinotecan and topotecan.

**Table 1.** Various functions of autophagy in response to the clinically used topoisomerase I inhibitors.

Compound	Cancer type/ cell line	Autophagy Modulation	Autophagy function	References
Irinotecanmetabolite, SN-38	LOVO and HCT116, SW1116 colorectal cancer cell lines and the overexpressing mutant p53 SW480 and HT-29 colorectal cancer cell lines	3-MA, CQ, ATG5 siRNA	Non-protective	[40]
NA	*Irinotecan-resistant* LOVO colon cancer cell line	N/A	High level of basal autophagy in these cell line	[41]
Irinotecan metabolite, SN-38	HSC-4 and HSC-2 human oral squamous cell carcinoma cell line	3-MA, Baf A1	Cytotoxic in HSC-4, however; non-protective in HSC-2	[42]
Irinotecan	MGC803 and SGC7901 gastric cancer cells lines	3-MA, CQ, Beclin1 siRNA	Cytotoxic	[36]
Irinotecan metabolite, SN-38	MDA-MB-231 and MDA-MB-436 triple-negative breast cancer cell lines	Baf A1, CQ, toosendanin (TSN)	Cytoprotective	[43]
Irinotecan metabolite, SN-38	HCT116-TP53 KO colon cancer cell line	Baf A1, 3-MA, ATG5 siRNA, ATG7 siRNA	Cytoprotective	[44]
Topotecan	A549 non-small cell lung cancer cell line	CQ, ATG5 siRNA	Cytoprotective	[45]
Topotecan	P53 wild-type (HCT116 and LS-174T) and p53 mutant (HT29, SW620, HCT116 p53^−/−^ and SW480) human colon cancer cell lines	Beclin1 siRNA, ATG-5 siRNA, CQ	Cytoprotective	[51]
